# miRNA–mRNA Conflux Regulating Immunity and Oxidative Stress Pathways in the Midgut of Blood-Fed *Anopheles stephensi*

**DOI:** 10.3390/ncrna1030222

**Published:** 2015-11-19

**Authors:** Shanu Jain, Jatin Shrinet, Adak Tridibes, Raj K. Bhatnagar, Sujatha Sunil

**Affiliations:** 1 Insect Resistance Group, International Centre for Genetic Engineering and Biotechnology, New Delhi 110067, India; E-Mails: shanujain07@gmail.com (S.J.); jatbioinfo@gmail.com (J.S.); 2 National Institute of Malaria Research, Dwarka, New Delhi-110077, India; E-Mail: adak.mrc@gmail.com

**Keywords:** *Anopheles*, Blood feeding, miRNA, Targets, Degradome, Co-Express, Lock nucleic acid, Oxidative stress

## Abstract

Blood feeding in *Anopheles stephensi* initiates a cascade of events that modulate several physiological functions in the mosquito. The midgut epithelium activates several of its molecules, most important among these being microRNAs, which regulate some of the physiological changes by targeting diverse mRNAs. The present study was conducted to identify and evaluate interactions between targets of eight miRNAs that are regulated on blood feeding. Identified from our previous study, we show these eight miRNAs exhibited distinct tissue specific expression. Targets of these miRNAs were predicted using computational approaches involving bioinformatics, co-expression analysis of the transcriptome and miRNome of blood-fed *An. stephensi* midgut. Using degradome sequencing, we identified some cleaved mRNAs of these microRNAs and, by using antagomiR knockdown technology to repress the miRNAs, the targets were validated in an *An. stephensi* cell line and in *An. stephensi* mosquitoes. In-depth analysis of predicted and identified targets revealed that the regulated miRNAs modulate well-characterized molecules that are involved in combating oxidative stress and immunity pathways through a dynamic miRNA:mRNA network. Our study is the first to identify miRNA:mRNA interactomes that play important role in maintaining redox homeostasis during blood feeding in the midgut of *An. stephensi*.

## 1. Introduction

Disruption of prooxidant-antioxidant balance in cells due to cellular respiration or immune response to invading pathogens causes oxidative stress [1,2]. Levels of reactive species, such as reactive oxygen species (ROS) and reactive nitrogen species (RNS), increase in the cells, damage DNA and proteins, and trigger apoptosis [3].

Hematophagus insects, such as mosquitoes, ingest blood meal to fulfill their nutritional requirement for energy and egg development. Blood digestion induced metabolic changes reduce ROS level and as a consequence impact metagenome status [4]. Such decrease in ROS level and flow of digested nutrients favors the proliferation of gut microbiota that maintains symbiotic relationship with the mosquito [4]. Perturbations in metagenome constituents are challenged by mosquito immune status to block proliferation of pathogen [5,6]. Digestion of blood protein, hemoglobin also results in release of highly reactive products, such as heme and iron. Free radicals are produced by excess iron that mediates oxidative damage to the mosquito cells [7]. Heme has a pro-oxidant and cytotoxic effect and its interaction with ROS generated in midgut could increase ROS toxicity [8,9]. To counter oxidative stress caused by various sources post blood feeding, hematophagus insects have evolved various mechanisms such as increased expression of antioxidant enzymes and heme aggregation and degradation [10,11,12]. The number of malaria parasite ingested along with blood meal dwindle due to excessive oxidative stress and innate immunity in the midgut of female mosquito [13]. Along with the insect vector, *Plasmodium* has also evolved defense mechanisms to protect against oxidative damage and transmit to next mammalian host [14,15,16].

Regulation of protein coding RNAs at post-transcription level is mediated by a class of small non-coding RNAs, known as miRNAs. MicroRNAs (miRNAs) play role in various physiological conditions as they bind to various gene sequences and regulate its expression, either by transcript decay and translational repression [17,18,19,20]. Different computational and experimental methods have been applied to identify miRNA targets [21,22]. Target identification based on miRNA binding to mRNA sequence using various computational tools has high probability of predicting false positive targets due to short miRNA sequence. Such shortcomings can be overcome by combining computational analysis with experimental data such as joint analysis of miRNA and mRNA transcriptome profiling generated in specific tissue of an organism. Such analysis would facilitate identification of correlating miRNA mRNA pairs interacting in spatio-temporal manner in an organism. Hence, miRNA-mRNA interacting pairs identified by different approaches and their network generation can be used to gain better insights into their role in various biological processes [23,24,25]. MicroRNAs have been identified in various mosquito species [26,27,28]. Blood feeding, as well as infection with pathogens, results in regulation of miRNA expression in specific tissues of host [29,30]. Few of these miRNAs have been characterized functionally, but very little has been known about probable function of miRNAs in the midgut of blood-fed female mosquito [31,32].

In this study, we examined miRNA:mRNA interactomes in the midgut of blood-fed *Anopheles stephensi* mosquitoes. Significantly regulated miRNAs from our previous study were profiled in the mosquito tissues post blood-feeding [26]. Targets of miRNAs expressed in midgut tissue were predicted using different approaches. Targets functional in immune pathways and redox detoxification pathways were validated using loss of function strategy by antagomir injections. Such miRNA-mRNA interactions help us to understand regulation of mosquito responses in midgut tissue, which might function to challenge pathogen invasion and development.

## 2. Results

**Figure 1:**
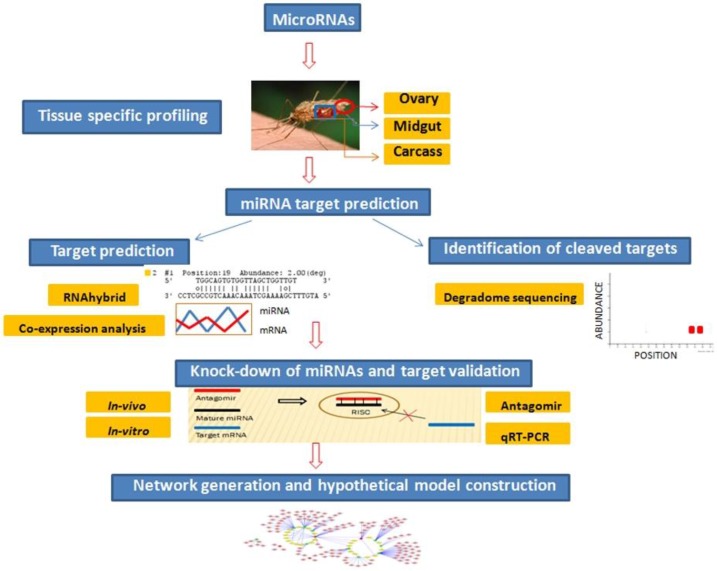
Flowchart to depict step-wise experimental analysis carried out to identify and validate miRNA targets functional in oxidative stress and innate immunity pathways in midgut tissue of female mosquito.

In our previous study, we identified miRNAs differentially expressed in whole body of blood-fed and infected mosquito [21] and this present study was undertaken to substantiate some of those miRNAs regulated upon blood-fed. For performing tissue specific profiling, we selected eight differentially expressed miRNAs (miR-34, miR-989, miR-277, miR-1174, miR-309, miR-285, miR-210, and miR-219) showing regulation upon blood feeding. Of these, three miRNAs, namely, miR-309, miR-285, and miR-210 were found to be absent in midgut, and miR-219 did not show any expression in the ovary. Five miRNAs (miR-34, miR-989, miR-277, miR-1174, and miR-219) that expressed in midgut tissue were selected to understand their role in innate immunity and oxidative stress in female mosquito. To this end, validation and prediction of miRNA targets was carried out using various approaches (Figure 1).

Preliminary miRNA target prediction was carried out by RNAhybrid and miRNA:mRNA co-expression analysis. Targets cleaved by midgut expressing miRNAs were identified by degradome sequencing. Candidate miRNA targets playing role in mosquito oxidative stress and innate immunity were validated by miRNA knockdown studies using antagomirs. To gain better understanding of role of these midgut expressing miRNAs and their targets, an interaction network was generated. Based on our results, we propose a model highlighting involvement of identified miRNA:mRNA interactomes in regulating redox state and immunity in midgut tissue of female mosquito. Each of the above-mentioned findings are discussed in detail below.

### 2.1. Tissue Specific Profiling of Regulated miRNAs

**Figure 2:**
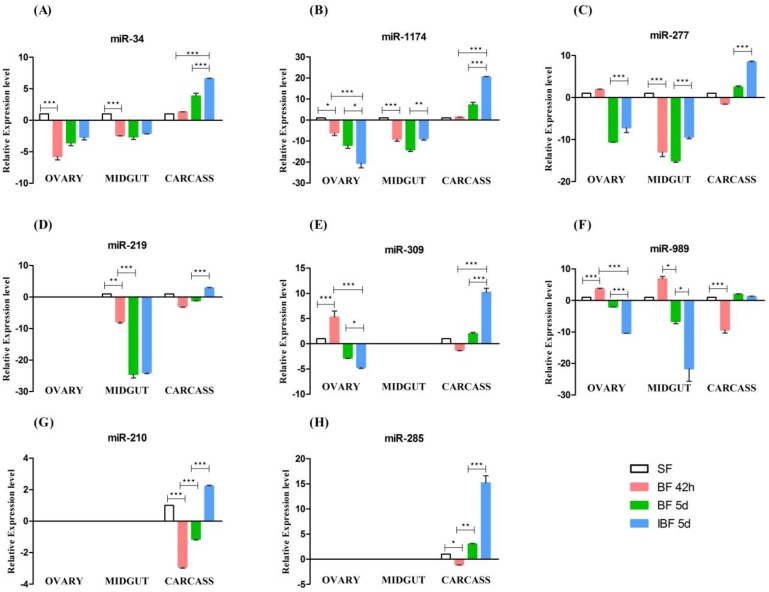
Tissue specific profiling of miRNAs by RT-PCR. Expression profiling of eight miRNAs, (**A**) miR-34; (**B**) miR -1174; (**C**) miR-277; (**D**) miR-219; (**E**) miR-309, (**F**) miR-989; (**G**) miR-210 and (**H**) miR-285 in ovary, midgut and carcass tissue of sugar fed (SF), female mosquito at 42 h (BF 42 h) and 5 days (BF 5d) post blood-feeding and mosquitoes at 5 days post *Plasmodium* infection (iBF 5d). Fold change was calculated using 2^−ΔΔC^_T_ method. SF was used as a calibrator, with its fold change taken as 1. Values are mean ± s.e.m of three biological replicates profiled in triplicate reactions. Data was statistically analyzed by one-way ANOVA followed by Tukey’s multiple comparisons test. * *p* < 0.05 was considered significant (** *p* < 0.01, *** *p* < 0.001).

The miRNAs were profiled in midgut, ovary, and carcass tissue of female mosquitoes at 42-h post blood-feeding (BF 42 h), female mosquitoes at five days post blood feeding (BF 5d) and female mosquitoes at five days post infected blood feeding (iBF 5d) mosquitoes. Four miRNAs (miR-34, miR-989, miR-277, and miR-1174) were expressed in all tissues, whereas the remaining four miRNAs (miR-309, miR-285, miR-210, and miR-219) did not show expression in one or two tissues. Out of these four miRNAs, miR-309 was absent in midgut, miR-219 in ovary, whereas miR-285 and miR-210 were found to be absent in both ovary and midgut tissues (Figure 2).

MicroRNAs miR-34, miR-1174, miR-277, miR-219, and miR-989 showed expression in midgut tissue of female mosquito (Figure 2A–D,F). We observed significant down-regulation of four miRNAs (miR-34, miR-1174, miR-277, and miR-219) in midgut of BF 42 h compared with sugar fed naive female mosquitoes six to eight days old (SF). At BF 5d, miR-34, miR-1174, and miR-219 miRNAs were further down-regulated in female midgut compared to BF 42 h. MicroRNA-989 levels down-regulates at BF 5d compared to BF 42 h (Figure 2F). Infection with *Plasmodium* parasite and development of oocysts resulted in up-regulation of two miRNAs (miR-1174 and miR-277) in iBF 5d compared to BF 5d (Figure 2). miRNA-989 was significantly down-regulated in midgut tissue of infected mosquitoes when compared to the blood-fed mosquitoes (Figure 2F).

In the ovary, five miRNAs (miR-34, miR-989, miR-1174, miR-277, and miR-309) were expressed out of the eight miRNAs selected. MicroRNA-989 and miR-309 were up-regulated (Figure 2E,F) whereas miR-34 and miR-1174 were down-regulated in ovary of BF 42h compared to SF (Figure 2A,B). Parasite presence in blood resulted in differential expression of miRNAs in ovary tissue. MicroRNA-277 was up-regulated, whereas miR-1174, miR-989, and miR-309 were down-regulated in ovary of iBF 5d when compared to BF 5d (Figure 2A,B,E,F).

Mosquito bodies devoid of ovary and midgut were collected as carcass. We observed significant differences of miRNA expression in the carcasses of blood-fed and infected mosquitoes. MicroRNA-210 and miR-989 (Figure 2G,F) were down-regulated at BF 42 h compared to SF (Figure 2A). MicroRNA-34 expression was up-regulated at BF 5d when compared to BF 42 h female mosquito. Expression of miR-34, miR-1174, miR-277, miR-309, miR-219, miR-210, and miR-285 were up-regulated in carcass tissue in the presence of parasite infection when compared to normal blood-fed mosquito (Figure 2). Results of miRNA expression and regulation in different tissues post blood feeding and infection are summarized in Table 1.

**Table 1:** Expression and regulation of miRNAs in different tissues of mosquitoes. Regulation of miRNAs was considered significant with * *p* < 0.05 (** *p* < 0.01, *** *p* < 0.001). Expression and regulation of miRNAs was studied in ovary, midgut and carcass tissue of sugar fed (SF), female mosquito at 42 h (BF 42 h) and 5 days (BF 5d) post blood-feeding and mosquitoes at 5 days post *Plasmodium* infection (iBF 5d). Sample in parenthesis indicates the experimental condition with which expression was compared to in a particular tissue of mosquito.

S.No	miRNA Name	Expressed in Tissues			Regulated in Ovary		Regulated in Midgut		Regulated in Carcass	
		Ovary	Midgut	Carcass	BF 42 h	iBF 5d	BF 42 h	iBF 5d	BF 42 h	iBF 5d
1	miR-34	Yes	Yes	Yes	*** (SF)		*** (SF)			*** (BF 42 h), *** BF 5d)
2	miR-1174	Yes	Yes	Yes	* (SF)	*** (BF 42 h), *** (BF 5d)	*** (SF)	** (BF 5d)		*** ( BF 42 h), *** BF 5d)
3	miR-277	Yes	Yes	Yes		*** (BF 5d)	*** (SF)	*** (BF 5d)		*** (BF 5d)
4	mir-219		Yes	Yes			*** (SF)			*** (BF 5d)
5	mir-309	Yes		Yes	*** (SF)	*** (BF 42 h), *(BF 5d)				*** (BF 42h), *** BF 5d)
6	mir-989	Yes	Yes	Yes	*** (SF)	*** (BF 42 h), *** (BF 5d)		* (BF 5d)	*** (SF)	
7	miR-210			Yes					*** (SF)	*** (BF 5d)
8	miR-285			Yes					* (SF)	*** (BF 5d)

### 2.2. RNAhybrid Based Prediction of miRNA Targets

miRNAs regulate gene expression by binding to target gene sequences. To decipher the role of midgut expressing miRNAs, it was essential to identify mRNAs with miRNAs binding site on their gene sequence. For this purpose we predicted putative targets of miRNAs on 3' UTR, as well as 5' UTR+ coding region of *An. stephensi* genes using RNAhybrid. Transcripts with perfect complementarity with miRNA seed region, *p* value < 0.05 and minimum binding energy of <−20 kcal/mol were selected as miRNA targets. Maximum number of targets (*n* = 5090) was identified for miR-34. Out of 5090, 576 were identified on the 3' UTR of the target genes. The remaining 4514 were found on 5' UTR + coding regions on the genes. Minimum targets (*n* = 178) were predicted for miR-1174, 34 of which were present on 3' UTR of mosquito genes. Target prediction for remaining miRNAs, miR-219, miR-277, and miR-989 resulted in prediction of 498, 238 and 1555 targets, respectively (Table S1).

### 2.3. MicroRNA: mRNA co-Expression Analysis

**Figure 3:**
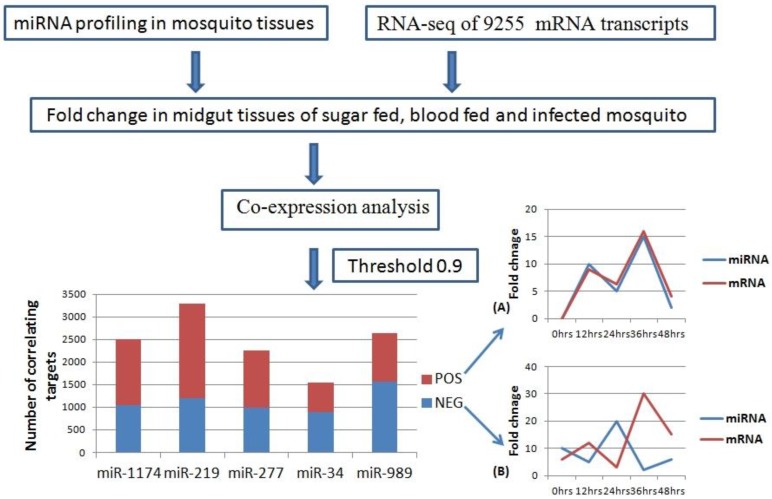
Workflow of miRNA and mRNA co-expression analysis. MicroRNA expression profile in mosquito midgut generated by real time profiling was taken as input data. RNA-Seq data of 9255 transcripts was used as second input data set. These two data sets were used to analyze miRNA: mRNA co-expression (Threshold = 0.9) in the midgut of the female mosquito. Bar graph depicts number of mRNAs showing positive (Red) and negative (Blue) correlation with miR-1174, miR-219, miR-277, miR-34, and miR-989, respectively in the mosquito midgut. (**A**) Line graph showing positive correlation between miRNA and mRNA over the time period of 0–48 h; (**B**) Line graph showing negative correlation between miRNA and mRNA over the time period of 0–48 h.

We carried out pair-wise correlation analysis of expression profiles of miRNAs and mRNAs using the CoExpress software tool [33]. Expression profiles of five miRNAs (miR-34, miR-1174, miR-277, miR-219, and miR-989) expressed in the midgut of female mosquito was used for the analysis. This data was correlated with expression levels of 9255 mRNA transcripts of *An. stephensi* generated using same midgut samples [34]. Transcripts showing both negative and positive correlation with miRNAs and threshold cut off of 0.9 were selected for further analysis (Table S2). Negatively correlated transcripts have expression pattern inversely proportional to the miRNA expression pattern (Figure 3). Whereas, positively correlated transcripts show the expression pattern same as that of miRNA targeting them (Figure 3). Total positively correlated events (*n* = 6535) were found to be more than total negatively correlated events (*n* = 5671) (Figure 3). Maximum number of positive and negative correlated transcripts was identified for miR-219 (*n* = 2077) and miR-989 (*n* = 1564) respectively. Minimum number of both positive (*n* = 654) and negative (*n* = 882) correlated transcripts was identified for miR-34 (Figure 3, Table S2).

Perfect correlated transcripts (threshold of ± 1) were identified for all the miRNAs. A total of 69 perfect negative correlated (threshold= (−1)) events were identified for five miRNAs, whereas 84 events were positively correlated (threshold = (+1)) with the miRNA expression profiles (Table S2). For miR-1174 and miR-989, set of perfectly correlated genes were enriched for genes belonging to cluster “protein binding” (GO:0005515). Genes perfectly correlated with miR-219 were enriched in cluster “protein binding” (GO:0005515) and “integral component of membrane” (GO:0016021).

To increase the stringency of target selection, miRNA targets predicted by both co-expression analysis and RNA hybrid were selected for further analysis. We identified total of 1004 mRNAs that were predicted as miRNA target in both the analysis. Number of shortlisted targets for miR-34, miR-1174, miR-219, miR-277 and miR-989 were 575, 29, 107, 28, and 265, respectively (Table S3). These shortlisted mRNAs have increased probability of being a *bona fide* miRNA targets as they showed co-expression pattern with specific miRNA and also possessed miRNA binding site on their sequence (Table S3).

### 2.4. Knockdown of miRNA Expression *in Vitro* and *in Vivo* Using Antagomirs

**Figure 4:**
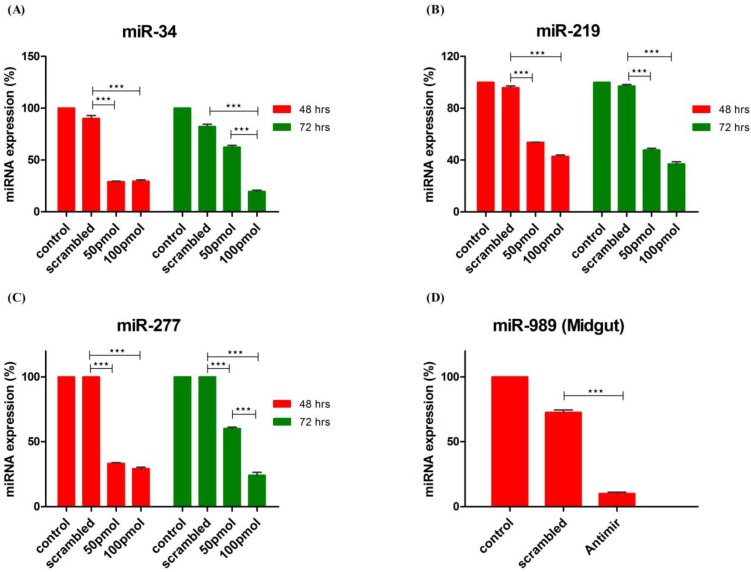
Knockdown of miRNA expression by antagomirs. Knock-down of (**A**) miR-34; (**B**) miR-219 and (**C**) miR-277 expression in cells transfected with 50 pmol and 100 pmol of antagomirs. MicroRNA expression was profiled by RT-PCR at 48 h and 72 h post-transfection in control, scrambled RNA and antagomir transfected cells. Control transfected cells were used as a calibrator, in which expression (%) of miRNA was taken as 100; (**D**) Knock-down of miR-989 by nano-injecting miR-989 specific antagomir in female mosquito. MicroRNA expression was profiled in midgut tissue of PBS, scrambled and antagomir injected female mosquito taking PBS injected mosquitoes as calibrator. Expression (%) of miRNA in PBS injected midgut tissue was taken as 100. Values are mean ± s.e.m. of three biological replicates profiled in triplicate reactions. Data was statistically analyzed by one-way ANOVA followed by Tukey’s multiple comparisons test. * *p* < 0.05 was considered significant (** *p* < 0.01, *** *p* < 0.001).

Efficiency of miRNA knockdown in cells by antagomirs was checked at two different time points *viz*. 48 h and 72 h post-transfection using two different antagomir concentration. Administering 50 pmol and 100 pmol concentrations of antagomir resulted in more than 50% knockdown of miRNA expression. Additionally, optimum knockdown was observed from 48 to 72 h post antagomir transfection. *An. stephensi* cell line was transfected with antagomirs specific for miR-34, miR-277 and miR-219, respectively. Control and Scrambled RNA transfected cells were used as control to ensure antagomir specific knockdown of miRNA expression. Knock-down of miRNA expression was checked at both time points using poly A tailed miRNA RT-PCR. Significant knockdown of all three miRNAs was observed post transfection with antagomir when compared to scrambled RNA and neat transfected cells. MicroRNA-34 expression decreased >60% at 48 h with both concentrations of antagomir used. At 72 h post-transfection, 100 pmol of antagomir resulted in decrease of miR-34 expression by >70% whereas 50 pmol antagomir concentration failed to sustain significant knock-down of miR-34 expression as observed at 48 h post-transfection (Figure 4A). MicroRNA-219 expression decreased >50% post antagomir transfection at both 48 and 72 h (Figure 4B). Expression of miR-277 decreased >60% with both concentrations of antagomir at 48 h post transfection whereas at 72 h significant decrease in expression was observed with 100 pmol of antagomir (Figure 4C).

MicroRNA 989 expression was significantly higher in female mosquito when compared to male mosquito [30]. Further, its expression was significantly up-regulated in whole body of female mosquito post blood-feeding [26]. In this study, we observed that blood-feeding causes its regulation in midgut tissue of female mosquito. These observations highlight impact of miR-989 in midgut of female mosquito that is important for metabolic events involving blood digestion, nutrient absorption or pathogen invasion. Hence, to understand its role in female mosquitoes post blood-feeding, we conducted *in vivo* knockdown of miR-989 expression to investigate its role in midgut tissue of female mosquitoes. MicroRNA-989 expression was knocked down by injecting miR-989 specific antagomir in female mosquitoes. Mosquitoes injected with scrambled RNA and PBS were considered as control to ascertain specific knockdown of miR-989 by antagomir. Injection with miR-989 specific antagomir resulted in knockdown of miRNA expression by >80% and >60% when compared to phosphate-buffered saline (PBS) and scrambled RNA injected mosquito midgut respectively (Figure 4D).

### 2.5. MicroRNA-989 Specific Regulation of mRNA Targets in Midgut of Female Mosquito

RNA isolated from midguts of PBS, scrambled RNA and antagomir injected blood-fed mosquito were used for degradome libraries preparation. Analysis of degradome library revealed seven mRNAs that were cleaved by miR-989 in midgut of PBS injected mosquito. Regulation of these mRNAs was studied in scrambled RNA and antagomir injected midguts to establish role of these targets in midgut of mosquito. Out of seven mRNAs found regulated in PBS-injected mosquito midguts, degradome reads were identified for four mRNAs in antagomir treated mosquito midguts. Other three mRNAs were not cleaved by miR-989 in antagomir injected mosquito midguts. Gene ontology (GO) terms of miR-989 cleaved targets are provided in Table S4.

**Figure 5:**
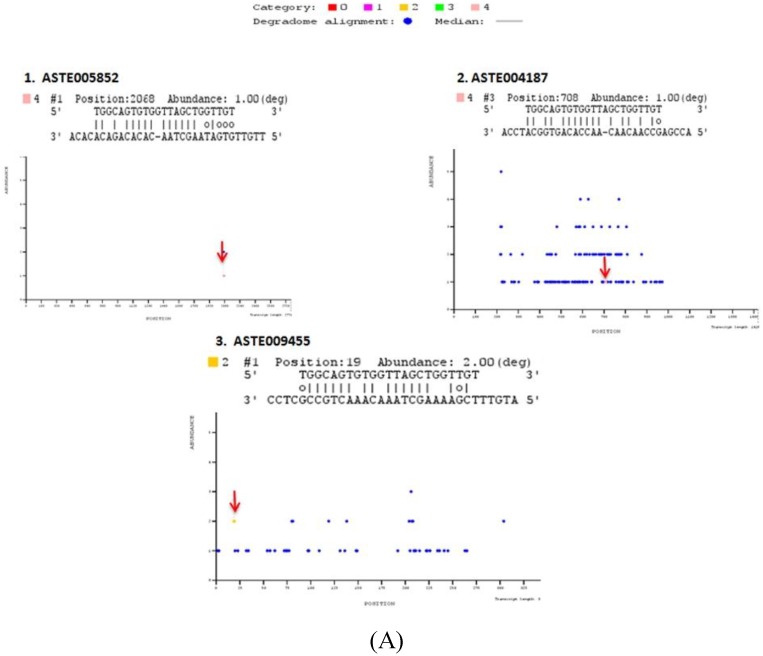

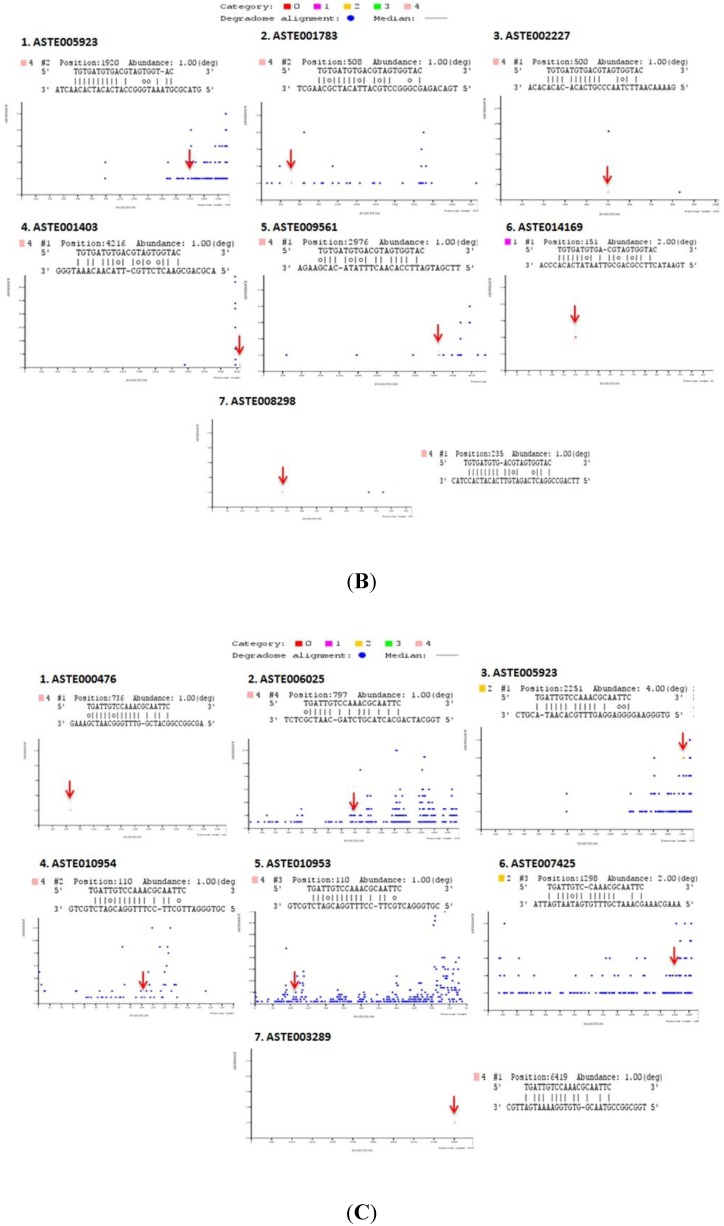
Degradome sequencing for identification of miRNA targets. Sequences reads are mapped on to the entire length of the transcript along with miRNA sequences using PAREsnip. T plots are generated showing abundance of mapped reads along the length of transcript. X-axis represents transcript length whereas Y-axis represents abundance of read mapped on to the specific position on the transcript sequence. Blue dot represents position mapped reads on transcript. Colored dots represent the position on the transcript where mapped degraded fragment (s) coincides with miRNA binding site (S). Identified miRNA targets are categorized in different categories (Category 0, 1, 2, 3, and 4), depending on their relative abundance on mapped position. Positions, where respective miRNA cleaves the transcript, are highlighted by an arrow. T-plots of (**A**) miR-34, (**B**) miR-989, (**C**) miR-219 degradome targets.

Our analysis revealed four other miRNAs (miR-34, miR-219, miR-277 and miR-1174) that were expressed in midgut tissue of female mosquito. mRNA targets cleaved by these miRNAs were also identified by degradome sequencing in PBS injected midgut tissue of female mosquito which served as a control in our experiments. We identified three and seven targets that were cleaved by miR-34 and miR-219, respectively (Table S4). MicroRNA-277 and miR-1174 did not cleave any target in midgut of female mosquito. Identified targets were classified into five different categories based on read abundance on the transcript sequence. Out of the total 17 targets identified, 13 were classified under category 4, having only one degradome read at the target cleavage site. Three targets were classified under category 2 with >1 read mapped on the position, the abundance is less than the maximum but greater than medium abundance for the transcript. Only one target was present under category 1 with >1 read at the position. Abundance of target reads at the mapped position equal to the maximum number of reads on more than one position on entire length of the transcript. (Table S4). T-plots of targets are shown in Figure 5.

Out of these 17 targets predicted by degradome sequencing, 11 targets were also predicted by either RNA hybrid or co-expression analysis or using both strategies (Table S4). Two targets, ASTE005923 and ASTE003289, were degraded by and have binding site(s) for miR-989 and miR-219, respectively, in their 3′UTR sequence. Five targets degraded by miR-34 (ASTE005852 and ASTE009455), miR-989 (ASTE014169), and miR-219 (ASTE010954 and ASTE010953), respectively, were also predicted to possess miRNA binding sites on 5′UTR or CDS region. Three targets predicted by co-expression analysis were also degraded by miR-34 (ASTE004187), miR-989 (ASTE008298) and miR-219 (ASTE007425). Only one transcript (ASTE002227) was identified to be degraded by miRNA, have binding site for miRNA in 3' UTR, and was co-expressed with miR-989 in midgut of female mosquito (Table S4). GO terms of these targets based on orthologs in *An. gambiae* are listed in Table S4. Prediction of these targets using different techniques highlights their significant function in the midgut tissue of an infected mosquito.

### 2.6. Validation of Targets Post Loss of Function of miRNAs in Mosquito Cells

**Figure 6:**
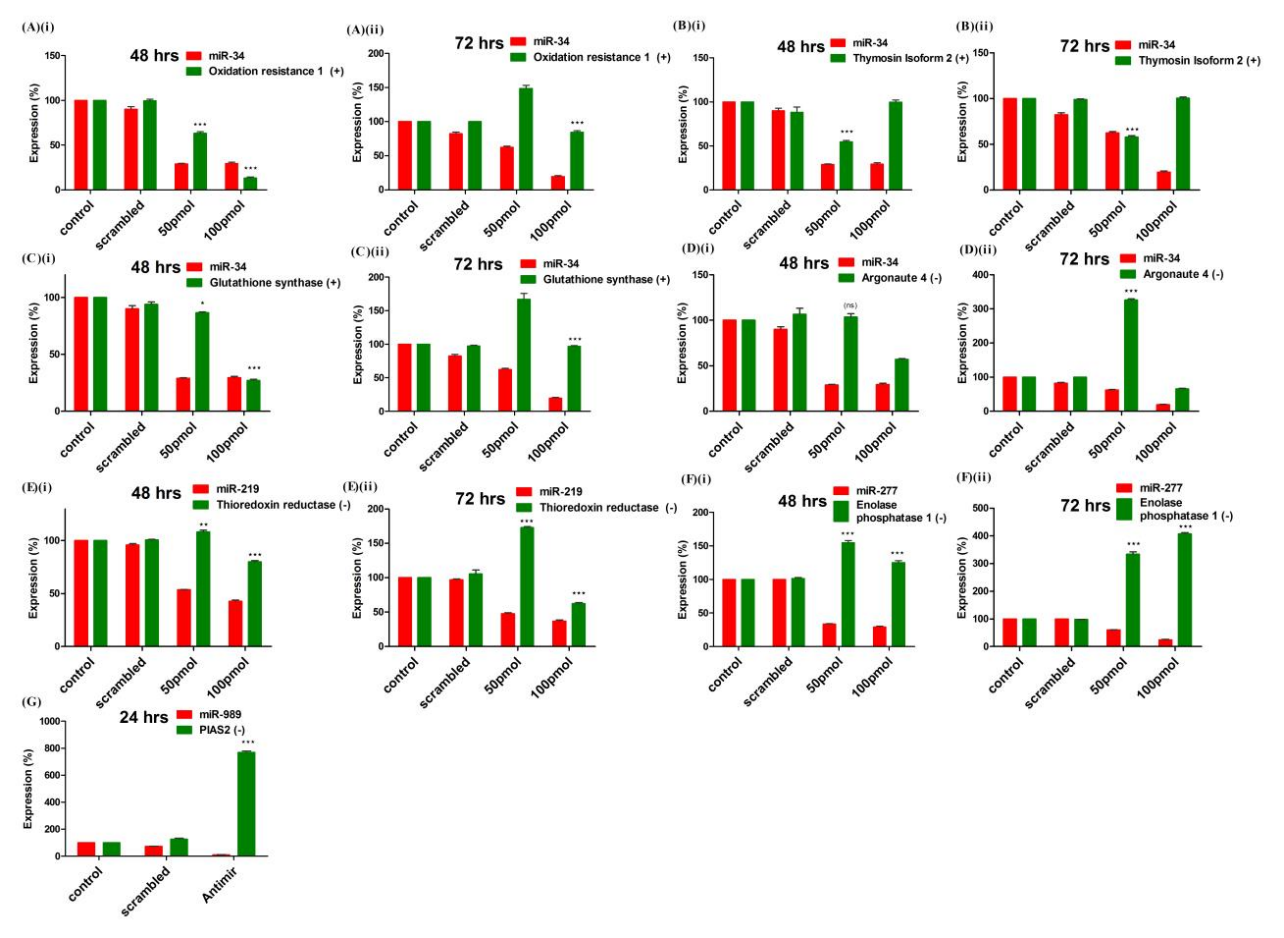
Validation of targets by miRNAs loss of function strategy: Expression profiling of mRNA target(s) of (**A**–**D**) miR-34; (**E**) miR-219 and (**F**) miR-277 was carried out by RT-PCR in control, scrambled and antagomir transfected cells. Expression (%) of mRNA was compared with miRNA expression at (**i**) 48 h and (**ii**) 72 h post-transfection; (**G**) MiRNA-989 target expression was profiled by RT-PCR in midgut of control, scrambled and miR-989 specific antagomir injected female mosquitoes. Expression (%) of miRNA and mRNA was compared at 24 h post blood-feeding. Expression (%) of mRNAs in control was taken as 100. Values are mean ± s.e.m of three biological replicates profiled in triplicate reaction. Data were statistically analyzed by one-way ANOVA followed by Tukey’s multiple comparisons test. * *p* < 0.05 was considered significant (** *p* < 0.01, *** *p* < 0.001). ns = No significant down-regulation.

A total of seven targets, functional in oxidative stress and innate immunity pathways, were selected for experimental validation. Expression pattern of mRNAs was quantified in miRNA knockdown cells using RT-PCR. Expression of four genes namely, glutathione synthase (ASTE007702), Thymosin isoform 2 (ASTE005472), Oxidation resistance protein (ASTE007150) and Argonaute 4 (ASTE005517) were quantified in miR-34 knockdown cells (Figure 6). Knock-down of miR-34 in antagomir transfected cells resulted in significant down-regulation of three genes (ASTE007150, ASTE005472 and ASTE007702), which were positively correlated with miR-34 in mosquito (Figure 6A–C). Cells transfected with 100 pmol of antagomir at both time points showed significant down-regulation of both miRNA expression, as well as positively correlated mRNA expression. Among the mRNA selected, Argonaute 4 (ASTE005517) was negatively correlated with miR-34 and, hence, its expression was not significantly regulated at 48 h post-transfection with 50 pmol of antagomir used (Figure 6D). At 72 h, Argonaute levels increased >300% with knockdown of miR-34 in transfected cells (Figure 6D).

Down-regulation of miR-219 and miR-277 resulted in up-regulation of negatively correlated targets, thioredoxin reductase (ASTE001205) and enolase phosphatase E1 (ASTE005393), respectively, in *An. stephensi* cell line (Figure 6E,F). Regulation of miR-989 targets was studied directly in the midgut tissue of female mosquito, nano-injected with miRNA specific antagomir (described in previous section). Knock-down of miRNA-989 resulted in up-regulation of protein inhibitor of activated STAT (ASTE009241) by >800%, a known agonist to *Plasmodium* development in midgut of a blood-fed female mosquito (Figure 6G).

### 2.7. miRNA:mRNA Interactomes Network Operating in the Midgut of Blood-fed Female Mosquito

**Figure 7:**
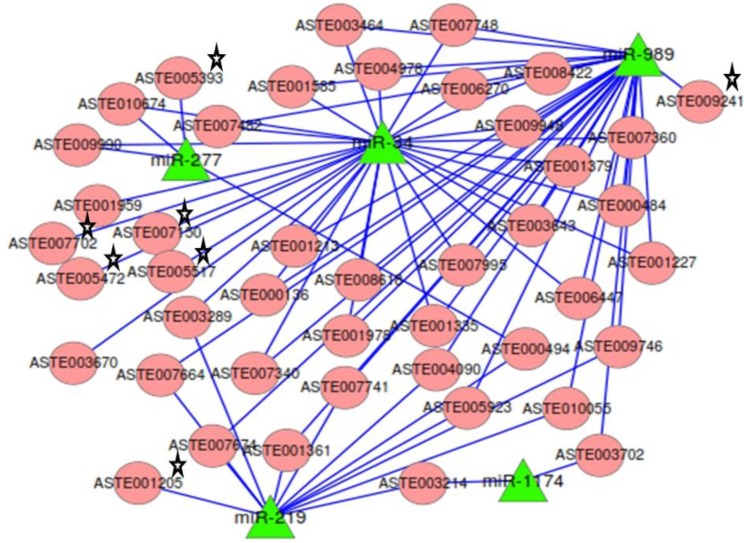
Network of miRNA:mRNA interactions. Network of five miRNAs expressed in the midgut tissue of female mosquito and their targets including the ones functional in oxidative stress and innate immunity. All miRNAs are depicted as a green colored node. Targets are depicted in circular and red colored nodes. Targets validated by miRNA loss of function are marked with stars.

Targets predicted by both RNAhybrid and co-expression analysis, along with degradome targets, were analyzed to identify the ones that were targeted by two or more miRNAs. mRNAs (*n* = 37) were targeted by two or more miRNA (Table S5). The network of miRNAs and targets functional in mosquito innate immunity and oxidative stress, along with 37 common targets, was generated (Figure 7). Among the 37 common targets, two cleaved mRNAs identified in degradome sequencing were targeted by more than one miRNAs. Target ASTE005923 was commonly degraded by both miR-219 and miR-989 and also contained binding site for miR-989 in its 3′UTR (Figure 7). It is annotated as acyl-CoA binding protein 2 in *An.gambiae*. Another target ASTE003289 was targeted by both miR-219 and miR-34. It functions as a rotatin protein and is degraded by miR-219 whereas it co-expresses and has binding site for miR-34 in its 3' UTR sequence. Many more targets of significant importance were identified as having binding site and showing co-expression with more than one miRNAs. One such target is ASTE004090, a Ras GTPase-activating protein 3 functional in MAPK signaling pathway, and is targeted by miR-219 and miR-989. Programmed cell death protein ASTE008618 was targeted by miR-34 and miR-989 (Figure 7). A protein functional in innate immunity, clip-domain serine protease, ASTE003643 was targeted by miR-34 and miR-989. Two transcripts, ASTE010674 and ASTE009990, functional in oxidative phosphorylation and endocytosis pathways, were targeted by both miR-34 and miR-277. Genes playing significant role in oxidative stress, such as glutathione synthase (ASTE007702), Oxidation resistance protein (ASTE007150), and Thioredoxin reductase (ASTE001205), were found to be targeted by these miRNAs. Argonaute (ASTE005517), an important component of RNAi machinery, was also found to be targeted by miR-34 in *An. stephensi* (Figure 7).

## 3. Discussion

Process of blood feeding causes regulation of miRNA expression in whole body of *Anopheles* female mosquitoes [26]. Tissue specific profiling of regulated miRNAs enhanced understanding of their expression post blood feeding in female mosquitoes. Four miRNAs were expressed in all the tissues profiled viz. midgut, ovary and carcass tissue of mosquito. Whereas, four other miRNAs showed tissue specific expression with their expression absent in midgut, ovary or both tissues of female mosquito. Further, blood feeding resulted in regulation of miRNAs in midgut and ovary of mosquito. Such blood feeding induced miRNA regulation highlights towards specific role of miRNAs in midgut tissue of female mosquito.

Digestion of haemoglobin in mosquito midgut lumen releases heme and iron that are pro-oxidant and cytotoxic to host cells. Oxy-haemoglobin and heme released throughout the RBC’s digestion react with NO and ROS to produce toxic metabolites [35]. Released heme decreases ROS levels locally in the midgut of female mosquito, which favors proliferation of microbiota immediately post blood meal [4]. Expanding midgut microbiota contributes to ROS levels in midgut tissue and activates basal immunity that causes enhanced expression of antimicrobial immune genes in the insect [5,36]. All these mechanisms, triggered post blood feeding, increase oxidative stress in mosquito body, especially in the midgut epithelia, the first tissue that gets exposed to blood and proliferating microbiota. Mosquitoes have adapted to increasing oxidative stress, causing high mortality by enhancing expression of antioxidant and detoxification enzymes post blood feeding [10,11]. Therefore, understanding the regulation of oxidant-antioxidant system and innate immunity in female midgut tissue post blood feeding is essential as these pathways can determine mosquito vectorial capacity to invading pathogens, such as malaria parasite.

**Figure 8:**
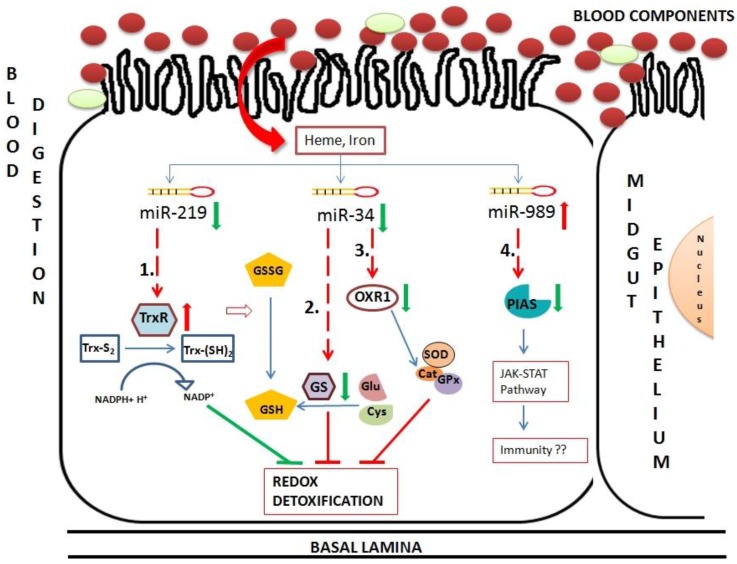
Hypothetical model of oxidative and immunity pathways regulated by miRNAs in midgut tissue of female mosquito post blood feeding. This pathway includes three miRNAs regulated in midgut of blood-fed female mosquito namely miR-34, miR-219 and miR-989. These miRNAs regulate expression of mRNAs, such as (1) Thioredoxin reductase (TrxR); (2) Glutathione synthase (GS); (3) Oxidation resistance 1 (OXR1) and (4) Protein inhibitor of activated STAT (PIAS). Red arrows and green arrows represent up-regulation and down-regulation of the component in midgut cells, respectively. Dotted lines represent miRNA:mRNA interaction validated in this study.

In this study, interactomes of midgut expressing miRNAs and their mRNA targets were deciphered to understand their role in maintaining redox state and immunity in mosquito midgut epithelium post blood feeding. Preliminary miRNA targets prediction was carried out using RNAhybrid and correlation analysis. mRNAs cleaved by miRNAs in midgut tissue were identified using degradome sequencing. We validated seven miRNA–mRNA interactions by knockdown of miRNA expression by antagomirs. These targets were annotated to be functional in pathways related to redox detoxification and innate immunity of mosquitoes. A component of RNAi machinery, Argonaute expression was regulated by miR-34 in mosquitoes. Regulation of Argonaute by miRNA highlight towards feedback regulation of total miRNA expression affecting various processes including the ones involved in stress and immunity in insects. Three other targets validated are known agonist or antagonist for *Plasmodium* development in mosquito. Oxidation resistance 1 (OXR 1) gene targeted by miR-34 regulates expression of detoxification enzymes and its silencing decreases *Plasmodium* oocyst formation [37]. Thymosin isoform 2 regulated by miR-34 act as a antagonist for *Plasmodium* development in the mosquito [38]. β Thymosin proteins bind to actin filaments and regulate actin-cytoskeletal dynamics. Various pathogens exploit such cytoskeleton proteins to facilitate their entry and exit from the host cells [39,40]. We validated targets of miR-989 by directly injecting antagomir in female mosquito. Knockdown of miR-989 expression resulted in regulation of Protein Inhibitor of Activated STAT (PIAS) in midgut tissue of blood-fed female mosquito. PIAS inhibits component of JAK-STAT pathway and, hence, its silencing decreased parasite numbers in the female mosquito [41]. Using validated miRNA–mRNA interactomes we proposed a model to unravel role of these interactomes in midgut tissue of blood-fed female mosquito (Figure 8). Insects possess thioredoxin and glutathione redox detoxification pathways to overcome oxidative stress generated by various factors including blood feeding. Blood feeding resulted in down-regulation of two miRNAs, miR-34 and miR-219 in midgut tissue. These miRNAs targets components of redox detoxification pathways and regulates their expression. Down-regulation of miR-219 resulted in up-regulation of its target, thioredoxin reductase. Reduced thioredoxin acts as an electron donor and reduce various oxidants. Hence, high levels of thioredoxin reductase work towards reducing damage caused by ROS toxicity post blood feeding. Down-regulation of miR-34 causes down-regulation of two targets, glutathione synthase and OXR1. Glutathione synthase is a major enzyme in glutathione biosynthetic pathway. Down-regulation of this enzyme results in deficiency of glutathione (GSH), required for ROS detoxification in cells. OXR1 regulates expression of major detoxification enzymes such as catalase, SOD and glutathione peroxidase [37]. Down-regulation of OXR1 results in down-regulation of these detoxification enzymes, increasing level of free radicals in the cells. Regulation of both glutathione synthase and OXR1 by miR-34 impairs the detoxification system of midgut cells post blood feeding (Figure 8).

Hence, we propose overall impairment of midgut detoxification system by validated miRNA-mRNA interactomes, which might be detrimental to invading pathogens. Further, PIAS regulated by miR-989 is a potential candidate that might play a major role in insect immunity. An in depth experimental validation is necessary to understand its role in midgut tissue of female mosquito.

## 4. Conclusions

This is a comprehensive study providing insight into the miRNA:mRNA interactomes in the midgut of female mosquito. Midgut of female mosquito is the primary tissue that gets exposed to blood, gut microbiota and invading pathogens. Efforts were concentrated on understanding regulation of targets functional in oxidative stress and innate immunity by mosquito miRNAs. Our analysis provides direct evidence about regulation of genes with the potential of altering oxidative stress in midgut of mosquito. These midgut miRNA:mRNA interactions can have consequences for the biology of disease vector and influence pathogen transmission, such as malaria parasites.

## 5. Material and Methods

### 5.1. Ethics Statement

Animal experiments were performed in accordance with National animal ethics guidelines of the Government of India after approval by Institutional Animal Ethics Committees of International Centre for Genetic Engineering and Biotechnology, New Delhi (Permit number: ICGEB/AH/2011/01/IR-8).

### 5.2. Insect Rearing, Blood Feeding and Infection

*An. stephensi* were reared at 28 ± 2 °C, 70%–75% humidity. Adults were fed on raisin and sterile glucose solution. 5–6 days old female mosquitoes were fed on uninfected and infected mice (*Plasmodium vinckei petteri* 279 BY) with 0.05% gametocytemia. Midgut of mosquitoes were dissected on 5^th^ day post infection and stained with mercurochrome to observe the presence of oocysts. Presence of oocysts in midgut confirmed infection of mosquitoes by the parasite.

### 5.3. Nano-Injections in Mosquitoes

Antagomir for miRNA was synthesized complementary to mature miRNA sequence with 2'-O-methyl (2'-OMe) group at each base and also with 3' cholesterol group (Dharmacon, Lafayette, CO, USA). Sequence of miR-989 antagomir is provided in Table S6. Scrambled RNA (Random sequence) was synthesized with same modifications and was used as a negative control. Four- to five-day-old female mosquitoes were knocked down on ice and around 100 were injected with 69 nL of 100 uM antagomir or scrambled RNA each. One hundred mosquitoes injected with 69 nL of PBS were taken as control in the study. Mosquitoes were allowed to recover for two days and were fed on uninfected mice blood. Midguts were dissected out of mosquitoes at 24 h post blood-feeding. Midguts were rinsed with PBS to wash away undigested blood bolus. Midguts were then stored in Trizol at −80 °C until RNA extraction. The experiment was replicated three times by injecting mosquitoes obtained from three different rearing cycles. Knockdown of miRNA expression in all three experiments was analyzed separately by miRNA qRT-PCR.

### 5.4. Tissue Dissection and RNA Preparation

Three different batches of mosquitoes were fed on normal and infected mice. Sugar fed, blood-fed and infected mosquitoes, 30 each, were collected from each batch and were processed separately. Dissection of tissues was performed under the microscope in PBS, pH 7.0 and tissues were transferred to Trizol solution kept on ice. Dissections were performed on 5–6 days sugar fed female mosquito. Midgut and ovary was dissected out and the remaining part of the body was then collected as carcass. Blood-fed mosquitoes were dissected at 42 h and 5 days post feeding whereas tissues of infected mosquitoes were collected at 5^th^ day post-infection. Midguts were cleaned of undigested bolus before collection. Total RNA from all the tissues were extracted using Trizol method (Invitrogen, Carlsbad, CA, USA) according to supplier’s instructions. RNA of tissues dissected from different batches were analyzed separately and were treated as biological replicates.

### 5.5. Maintenance and Transfection of An. stephensi Cell Line

*An. stephensi* was maintained in Schneider’s insect medium supplemented with 15% FBS and 1% pen-strep solution at optimum conditions of 27 °C with 5% CO_2_. Appropriate volume of cell suspension containing 10^6^ cells was seeded in each well of six well plates. Three milliliters of complete media was added to each well. Plates were rotated sideways to allow even distribution of cells over the entire surface of the well. Plates were incubated overnight at 27 °C with 5% CO_2_. Mosquito cell line was transfected with antagomirs using cellfectin reagent. Antagomirs were synthesized as described before. Sequences of miR-34, miR-219 and miR-277 antagomirs are provided in Table S6. Fifty picomole and 100 pmol of scrambled and miRNA specific antagomirs were mixed with 100 µL of serum free media in different eppendorfs. Ten microliter (10 µL) of cellfectin reagent was mixed with 90 µL serum free media. Media with cellfectin was added drop-wise to media containing scrambled and antagomirs. Plates were swirled gently and were incubated for 15 min at RT. Cells in 6 well plates were washed three times with serum free media. Two hundred microliter (200 µL) of solution containing cellfectin and RNA was added to each well of 6 well plates. Serum free media with cellfectin, devoid of RNA, was added to control wells. Plates were kept at 27 °C with slow rocking. After five hours, serum containing media was added to all the wells and cells were again incubated at 27 °C with 5% CO_2_. Cells were harvested at 48 h and 72 h post transfection and were stored in Trizol at −80 °C till RNA extraction. MicroRNA knockdown in cells was replicated three times in cells undergoing different passage cycle. Knockdown of miRNAs in each experiment was analyzed separately by RT-PCR.

### 5.6. Quantitative miRNA RT-PCR

Total RNA was quantitated and 10 ng of RNA was reverse transcribed to cDNA using universal cDNA synthesis kit (Exiqon, Vedbæk, Danemark). cDNA was 1:80 diluted and used in the reaction. PCR reactions were set up in triplicates with three biological replicates in ABI one step detection system using SYBR green master mix (Exiqon) following the manufacturer’s instructions. The mature miRNA sequence was used to synthesize custom miRNA LNA PCR primer sets (Exiqon). Ribosomal RNA(5.8 s r RNA) was used as an endogenous control for miRNA expression profiling as described before [26]. Fold change in expression was calculated using 2^−ΔΔC^_T_ method^ΔΔC^_T_^method^, taking SF as a calibrator for tissue specific profiling. Expression (%) of miRNAs was calculated using fold change values. MicroRNA expression (%) in scrambled and antagomir injected mosquito midgut was calculated using PBS injected midgut tissue as calibrator, expression of miRNA in which was taken as 100.

### 5.7. Quantitative Real Time PCR Profiling of mRNA Transcripts

mRNA expression was quantified in control, scrambled and antagomir transfected cells using QIAGEN One step RT-PCR kit. Fifty nanograms (50 ng) of total RNA was used per reaction. All reactions were set up in triplicates with three biological replicates in ABI one step detection system. S7 was used as an endogenous control. Primers with amplicon size of 120–150 nt were designed from the coding sequence of genes downloaded from the Vectorbase. Fold changes in transcript expression in scrambled and antagomir-transfected samples were calculated using 2^−ΔΔC^_T_ method with control RNA as calibrator. Expression (%) of mRNAs was calculated using fold change values. Sequences of gene primers are provided in Table S6.

### 5.8. miRNA Target Prediction by RNA Hybrid

RNA hybrid was used to predict multiple binding sites of miRNA on large target mRNAs [42]. 3' UTR, 5' UTR and coding region of *An. stephensi* genes were downloaded from vector-base and used as a reference sequence for target prediction [43]. Three parameters were used for target prediction (i) perfect complementarity of miRNA seed region on transcript sequences; (ii) *p* value < 0.05; and (iii) binding energy of miRNA on mRNA should be <−20 kcal/mol. Transcripts fulfilling all three criteria were selected as a target of specific miRNAs and were used for further analysis.

### 5.9. miRNA:mRNA co-Expression Analysis

Fold change in miRNA expression profile deduced by real time PCR was used for co-expression analysis. For mRNA expression data, we obtained RNA-Seq data generated using midgut tissues of sugar fed naive female mosquitoes 6–8 days old (SF), female mosquitoes at 5 days post blood-feeding (BF 5d), 5 days post *Plasmodium vinckei petteri* infected blood feeding (iBF 5d) and 5 days post human blood-fed (HBF 5d) mosquitoes [34]. Data were normalized (FPKM values calculated) and fold change of transcripts were calculated using SF as a calibrator. MicroRNA and mRNA expression data generated were used to identify correlated miRNA:mRNA pairs using Co-Express software [33] keeping the threshold as 0.9.

### 5.10. Degradome Sequencing for Identification of Cleaved miRNA Targets

Total RNA was extracted from midgut of mosquitoes post PBS, scrambled RNA and antagomir injections as described before. Degradome library construction and sequencing was performed by LC Sciences (Houston, TX, USA). Briefly, poly(A) RNA was enriched from 10 μg of total RNA using Oligotex mRNA mini kit (Qiagen, Venlo, The Netherlands). Degraded RNA fragments with 5'-monophosphate were ligated with 5' RNA adaptor using T4 RNA ligase. Ligated product was reverse transcribed using oligo (dT) primer with 3' adaptor sequence to generate first strand of cDNA via SuperScript II RT. The resulting cDNA was PCR amplified. The PCR amplified products were digested with Mme I (NEB, Evry, France) to generate equal sized products. A double stranded DNA was ligated to the MmeI digestion products using T4 DNA ligase. The ligated product was size fractionated on 10% polyacrylamide gel. Gel purified products were PCR amplified, purified and subjected to SBS sequencing. Raw reads, obtained after sequencing, were processed to remove low quality reads. Adaptor sequences were then trimmed from the raw reads. Resulting 20–25 nt sequences were selected for further analysis. Potentially cleaved miRNA target were identified using Parallel Analysis of RNA Ends (PAREsnip) [44]. Degradome sequences, cDNA of *An. stephensi* downloaded from Vectorbase and mature miRNA sequences were provided as an input to the PAREsnip software. Target plots (T-plots) were generated showing relative abundance of fragments mapping at the miRNA target site relative to the abundance of fragments found at other sites on the transcript. Depending on this, identified targets were grouped into five categories. In category 0, the maximum number of degraded sequences (>1) was present at one site on the transcript. In category 1, two or more sites were present on the transcripts where degraded sequences (>1) map with the same abundance. If abundance at a site was less than the maximum but greater than the median abundance for the transcript, the target was classified as category 2. In category 3, abundance at a position was less or equal to the median value for that transcript. Category 4 was classified with only one raw read at the position.

### 5.11. Network Generation of miRNA:mRNA Interactomes

Orthologues of *An. stephensi* gene IDs present in *An. gambiae* were fetched from vector-base database and their GO terms were used to assign functional to all miRNA targets identified. Significant targets were used to generate miRNA: mRNA interaction network. The resulting network was visualized using cytoscape [45].

### 5.12. Statistical Analysis

miRNA targets predicted using RNAhybrid were selected on the basis of significant *p* value ≤ 0.05 and minimum binding energy ≤−20 kcal/mol [46]. Targets were also predicted using CoExpress tool in which “between-experiment normalization” was performed to normalize the data and co-expression matrix was calculated for the data. The robustness of the co-expression matrix was validated by performing a bootstrapping step, as described elsewhere [33].

Statistical analysis was performed for real time PCR based expression profiling of miRNA and mRNAs using one-way ANOVA analysis of variance followed by Tukey’s multiple comparisons test. Values were presented as mean ± s.e.m. At *p* < 0.05, differences were considered significant.

## Supplementary Materials

**Table S1.** List of miRNA targets predicted by RNA hybrid with binding energy of <−20 kcal/mol and *p* value of <0.05.

**Table S2.** List of targets that were co-expressed with miR-34, miR-1174, miR-219, miR-277 and miR-989 respectively. Analysis was done using co-express software keeping threshold as 0.9.

**Table S3.** List of transcripts that were common between RNAhybrid and co-expression analysis. RNAhybrid was carried out to identify the miRNA binding site(s) on 3′UTR and on 5′UTR+CDS region of the transcripts.

**Table S4.** List of targets identified by degradome sequencing for miR-34, miR-989 and miR-219 in the midgut tissue of female mosquito.

**Table S5.** List of transcripts targeted by more than one miRNA. Column D and E list the miRNAs targeting these transcripts. Column F gives the GO term name for these transcripts depending on their orthologs in An.gambiae.

**Table S6.** List of antagomir and gene primers used in this study.
